# Mean Platelet Volume Reflect Hematopoietic Potency and Correlated Blood Group O in Cord Blood from Healthy Newborn

**DOI:** 10.1155/2013/754169

**Published:** 2013-03-27

**Authors:** Hye Ryun Lee, Jeong Su Park, Sue Shin, Eun Youn Roh, Jong Hyun Yoon, Eun Young Song, Byung Jae Kim, Ju Young Chang

**Affiliations:** ^1^Department of Laboratory Medicine, Gyeongsang National University Hospital, Gyeongsangnam-do, Jinju 660-702, Republic of Korea; ^2^Department of Laboratory Medicine, Seoul National University College of Medicine, Seoul 156-707, Republic of Korea; ^3^Department of Laboratory Medicine, Boramae Hospital, Seoul 156-707, Republic of Korea; ^4^Seoul Metropolitan Government Public Cord Blood Bank (Allcord), Boramae Hospital, Seoul 156-707, Republic of Korea; ^5^Department of Obstetrics and Gynecology, Boramae Hospital, Seoul 156-707, Republic of Korea; ^6^Department of Paediatrics, Boramae Hospital, Seoul 156-707, Republic of Korea

## Abstract

We evaluated the relationship between mean platelet volume (MPV) and characteristics of 10,577 cord blood (CB) units in a public CB bank in Korea. Blood group O has the highest MPV (*P* = 0.002). MPV correlated with CB volume (*r* = 0.121), Hb (*r* = 0.377), WBC (*r* = 0.111), TNCs (*r* = 0.110), CD34+ cell (*r* = 0.174), CD34+ cells/TNCs (*r* = 0.157), gestational age (*r* = −0.102), and birth weight (*r* = 0.023); (*P* < 0.001 in all). MPV may be one of the useful decision parameters of process priority in CB bank.

## 1. Introduction

Among CB units with similar TNC counts and HLA types, the CD34+ cell count is used to select the best unit [1–3]. To improve the quality of CB units in banks, robust and easy-to-perform tests are needed to predict hematopoietic potency and transplantation outcomes before processing and storing CB.

Mean platelet volume (MPV) has been reported to correlate with TNC and CD34+ cell counts, suggesting that MPV may be useful as a predictor of hematopoietic potency and transplantation outcomes [4]. Therefore, we retrospectively analyzed a large number of CB units to ascertain the relationship between MPV and hematopoietic parameters and other variables, including TNC and CD34+ cell counts, hemoglobin and platelet concentrations, WBC count, and maternal and neonatal factors. These values were also evaluated according to the ABO blood groups of the neonates.

## 2. Design and Methods

### 2.1. Subjects and Data Collection

Data from a total of 10,577 CB units that were processed and stored at the Seoul Metropolitan Public Cord Blood Bank (Allcord) from October 2006 through August 2009 were analyzed. The CB units were donated by pregnant Korean women who provided informed consent. The study protocol was approved by the Institutional Review Board of Seoul National University Boramae Hospital (06-2012-64). We used in utero technique from the term healthy infant without any perinatal problems. CB was collected into a collection bag containing 24.5 mL citrate phosphate dextrose adenine-1 as an anticoagulant. Within 24 hours after delivery, the collected CB was transferred to the CB bank in a shipping container at 15°C to 25°C to maintain cell viability according to the standard operating procedures of Allcord. The units containing more than 60 mL CB (excluding anticoagulant) were subject to be processed. The automated blood cell counter (XE-2100; Sysmex, Kobe, Japan) was used to determine the MPV and concentrations of Hb, WBCs, and platelets of all CB units. MPV was calculated by dividing the plateletcrit by the number of platelets. After volume reduction for cryopreservation, the blood was further tested to determine TNC and CD34+ cell counts and ABO blood group, according to the standard operating procedures of Allcord as described elsewhere [5]. 

Relationships between variables were determined by simple linear regression analysis. These variables were also compared according to ABO blood group by analysis of variance and the Kruskal-Wallis test. All statistical analyses were performed using the Statistical Package for Society Sciences (SPSS) (version 12.0; IBM Corp., Chicago, IL, USA); *P* < 0.05 was considered significant. 

## 3. Results and Discussion

Table 1 shows the characteristics of the mothers (age) and neonates (gestational age, birth weight, sex, mode of delivery, and ABO blood group).  Table 2 shows the following characteristics of the 10,577 CB units: volume, concentrations of Hb, WBCs, and platelets MPV from the units before volume reduction process and TNC count, CD34+ cell count, and CD34+ cells/TNCs from the units after the process. Analysis of the 10,577 CB units showed that MPV was positively correlated with CB volume (*r* = 0.121, *P* < 0.001), Hb concentration (*r* = 0.377, *P* < 0.001), and WBC count (*r* = 0.111, *P* < 0.001) but not platelet concentration. In addition, MPV was positively correlated with TNC count (*r* = 0.110, *P* < 0.001), CD34+ cell count (*r* = 0.174, *P* < 0.001), and CD34+ cells/TNCs (*r* = 0.157, *P* < 0.001). No relationship was found between MPV and maternal age (*r* = 0.003, *P* = 0.739). However, MPV was negatively correlated with gestational age (*r* = −0.102, *P* < 0.001) and positively correlated with birth weight (*r* = 0.023, *P* = 0.020). Neonatal gender was not associated with MPV. When we did the multivariate analyses of MPV with CB volume, CD34+ cell count, WBC count, TNC, gestational age, and birth weight, the associations were also significant (see Supplementary Table 1 available online at: http://dx.doi.org/10.1155/2013/754169). 

Table 3 shows the characteristics according to the ABO blood group. The birth weight of the neonates and CB values for Hb concentration, platelet concentration, and MPV differed according to ABO blood group. Although neonates with blood type O had the lowest birth weight (*P* for trend = 0.003), their CB units showed the highest MPV (*P* for trend = 0.002) and Hb concentration (*P* for trend < 0.001). In contrast, CB units from neonates with blood type A had the highest platelet concentration (*P* for trend < 0.001).

The present study shows that the MPV of CB correlates with TNC and CD34+ cell counts and the ratio of CD34+ cells to TNCs in the processed CB units, which is consistent with the results of previous studies [4, 6]. Our study is significant because of the large number of CB units analyzed from a Korean CB bank. 

We previously reported the relationship between ABO blood group and hematopoietic parameters (TNC count, CD34+ cell count, and CD34+ cells/TNCs) in CB units [5]. CB obtained from neonates with blood type O has higher TNC and CD34+ cell counts and ratio of CD34+ cells to TNCs than neonates with other blood types; therefore, CB units with blood type O may have greater hematopoietic potency. In this study, we also found that neonates with blood type O had significantly higher MPV. However, neonates with blood type A, not neonates of blood type O, had the highest platelet concentration.

In this study, we found that MPV appeared to be inversely correlated with platelet concentration in CB units, but this relationship was not statistically significant. Studies have reported a nonlinear inverse relationship between MPV and platelet count in the peripheral blood of adults [7, 8]. In contrast, a positive correlation has been reported between MPV and platelet concentration in CB units; however, that study analyzed only 167 units [4]. We analyzed a pretty much larger number of CB units in our study (10,577); therefore, we believe there is no proof of relationship between MPV and platelet concentration. Our finding that MPV positively correlated with Hb is consistent with that of a previous study [6]. MPV was also positively correlated with WBC concentration. 

MPV was not associated with maternal age or neonatal gender but was negatively correlated with gestational age and positively correlated with birth weight.

Anticoagulants can affect MPV in CB after collection. In particular, EDTA causes time-dependent platelet swelling, which increases MPV [9]. Therefore, it is important to use alternative anticoagulants and reduce the time between collection and analysis. Because MPV changes can be reduced using citrate-based anticoagulants [10, 11], we used collection bags containing the citrate-based anticoagulant CPDA-1. In addition, platelet clumping and early clotting is a concern after ex utero collection of CB. Therefore, we collected CB in utero to better evaluate the relationship between platelet concentration and MPV. 

In summary, this study reveals that the MPV of CB units correlates with TNC and CD34+ cell counts and the gestational age and birth weight of neonates. MPV is routinely measured with other blood components using automatic hematologic analyzer before CB processing but is not currently used as a predictor of hematopoietic potency. However, MPV values combined with CB volume limit of 60 mL before processing and TNC and CD34+ cell counts after processing may be useful in the selection of high-quality units for storage. 

## Supplementary Material

The result of multiple linear regression analysis.

## Figures and Tables

**Table 1:** Characteristics of mothers and neonates.

Variable	*n*	%	Mean ± SD	Median	Range
Maternal age (years old)	**10,577**	****	**30.8 ± 3.5**	**31.0 **	**16–49**
<20	20	0.2			
20–24	265	2.5			
25–29	3,561	33.7			
30–34	5,257	49.7			
35–39	1,363	12.9			
≥40	111	1.0			
Gestational age (weeks)	**10,577**		**39.1 ± 1.2**	**39**	**32–42**
<36	57	0.5			
36	165	1.6			
37	669	6.3			
38	2,136	20.2			
39	3,157	29.8			
40	3,361	31.8			
41	1,014	9.6			
42	18	0.2			
Birth weight (kg)	**10,577**		**3.36 ± 0.38**	**3.35**	**1.66–6.70**
≤2.50	118	1.1			
2.51–3.00	1,693	16.0			
3.01–3.50	5,349	50.6			
3.51–4.00	2,956	27.9			
>4.00	461	4.4			
Sex of neonates	**10,577**				
Male	5,456	51.6			
Female	5,121	48.4			
Mode of delivery	**10,571**				
V/D	7,781	73.6			
C/S	2,790	26.4			
ABO blood group of neonates	**10,577**				
A	3,669	34.7			
B	2,854	27.0			
O	2,844	26.9			
AB	1,210	11.4			

V/D: vaginal delivery; C/S: Cesarean section; SD: standard deviation.

**Table 2:** Characteristics of 10,577 cord blood units.

Variable	Mean ± SD	Median	Range
Volume (mL)	86.0 ± 18.0	83.0	53.9–201.2
Hb (g/dL)	11.7 ± 1.3	11.7	5.4–17.4
WBCs (× 10^3^/*μ*L)	10.9 ± 3.0	10.4	3.2–31.2
Platelets (/*μ*L)	207 ± 39	205	72–422
MPV (fL)	9.5 ± 0.6	9.4	7.6–13.2
TNCs (× 10^8^/unit)	8.8 ± 3.0	8.3	1.5–32.2
CD34+ cells (× 10^6^/unit)	1.15 ± 1.09	0.84	0.02–15.67
CD34+ cells/TNCs (%)	0.123 ± 0.089	0.101	0.007–1.102

Hb: hemoglobin; WBCs: white blood cells; MPV: mean platelet volume; TNCs: total nucleated cells; SD: standard deviation.

**Table 3:** Gestational age, birth weight, cord blood volume, blood cell components, mean platelet volume, and other hematopoietic parameters of the cord blood units according to ABO blood group of neonates.

	A	B	O	AB	*P*
	(*n* = 3669)	(*n* = 2854)	(*n* = 2844)	(*n* = 1210)	
Gestational age (weeks)	39.1 ± 1.2	39.1 ± 1.2	39.1 ± 1.2	39.1 ± 1.2	0.406
Birth weight (kg)	3.37 ± 0.38	3.35 ± 0.38	3.34 ± 0.37	3.37 ± 0.37	0.003
Volume (mL)	86.3 ± 18.2	85.6 ± 17.8	86.1 ± 18.2	86.0 ± 18.0	0.393
Hb (g/dL)	11.6 ± 1.3	11.7 ± 1.3	11.8 ± 1.3	11.6 ± 1.3	<0.001
WBCs (× 10^3^/*μ*L)	10.8 ± 3.1	10.9 ± 2.9	11.0 ± 3.1	10.8 ± 3.1	0.092
Platelets (/*μ*L)	209 ± 39	205 ± 38	206 ± 39	206 ± 38	<0.001
MPV (fL)	9.486 ± 0.607	9.446 ± 0.610	9.507 ± 0.617	9.466 ± 0.616	0.002

SD: standard deviation; Hb: hemoglobin; MPV: mean platelet volume; WBCs: white blood cells; TNCs: total nucleated cells.
