# Supplementary material for: Mean Platelet Volume Reflect Hematopoietic Potency and Correlated Blood Group O in Cord Blood from Healthy Newborn
Source: Biomed Res Int. 2013 Mar 27;2013:754169. doi: 10.1155/2013/754169 (PMC3625541; doi:10.1155/2013/754169)
Supplement: Supplementary file 1 — The result of multiple linear regression analysis. [file 754169.f1.docx]

(Supplementary Table 1)

**■ Multiple linear regression**

Response variable: MPV

Explanatory variables: CD34, CB Volume, WBC, TNC, GA, Birth wt

| **Coefficients^a^** | | | | | | | | |
| --- | --- | --- | --- | --- | --- | --- | --- | --- |
| Model | | Unstandardized Coefficients | | Standardized Coefficients | t | Sig. | Collinearity Statistics | |
|  |  | B | Std. Error | Beta |  |  | Tolerance | VIF |
| 1 | (Constant) | 11.063 | .202 |  | 54.734 | .000 |  |  |
|  | CD34 | .001 | .000 | .090 | 7.480 | .000 | .619 | 1.617 |
|  | Volume_CBU | .005 | .000 | .155 | 11.076 | .000 | .459 | 2.178 |
|  | WBC | .050 | .005 | .247 | 10.731 | .000 | .169 | 5.920 |
|  | TNC | -.039 | .005 | -.191 | -7.282 | .000 | .131 | 7.638 |
|  | GA | -.060 | .005 | -.115 | -11.548 | .000 | .898 | 1.114 |
|  | BirthWt_centile_code | .012 | .005 | .024 | 2.495 | .013 | .932 | 1.073 |
| a. Dependent Variable: MPV | | | | | | | | |
